# Association between elevated C-reactive protein and mortality, healthcare resource utilization and costs in patients with atherosclerotic cardiovascular disease and/or chronic kidney disease

**DOI:** 10.1093/ehjopen/oeag177

**Published:** 2026-10-14

**Authors:** Kausik K Ray, Fotios Barkas, Eline L Huisman, Jimmi Mathisen, Iram Faqir Muhammad, Hongye Ren, Simon Bertram Reuter, Jonathan Pearson-Stuttard, Andrew Thompson

**Affiliations:** Department of Primary Care and Public Health, School of Public Health, Imperial College London, 320 Reynolds Building, Charing Cross Campus, London, W6 8RP, UK; Department of Primary Care and Public Health, School of Public Health, Imperial College London, 320 Reynolds Building, Charing Cross Campus, London, W6 8RP, UK; Department of Internal Medicine, Faculty of Medicine, School of Health Sciences, University of Ioannina, 45110 Ioannina, Greece; Novo Nordisk BV, Kon. Wilhelminaplein 2c, 1062 HK Amsterdam, Netherlands; Novo Nordisk A/S, Vandtårnsvej 108, 2860 Søborg, Denmark; Novo Nordisk A/S, Vandtårnsvej 108, 2860 Søborg, Denmark; Novo Nordisk A/S, Vandtårnsvej 108, 2860 Søborg, Denmark; Novo Nordisk A/S, Vandtårnsvej 108, 2860 Søborg, Denmark; LCP Health, Lane Clark & Peacock LLP, 95 Wigmore Street, London, W1U 1DQ, UK; Department of Epidemiology and Biostatistics, School of Public Health, Imperial College London, St Mary’s Campus, Norfolk Place, London, W2 1PG, UK; LCP Health, Lane Clark & Peacock LLP, 95 Wigmore Street, London, W1U 1DQ, UK

**Keywords:** Atherosclerotic cardiovascular disease, C-reactive protein, Chronic kidney disease, Healthcare resource utilization, Inflammation, Mortality

## Abstract

**Aims:**

Atherosclerotic cardiovascular disease (ASCVD) and chronic kidney disease (CKD) impact health and healthcare systems. Inflammation is a potential modifiable risk factor for both conditions. The case for evaluating the modification of inflammation in these conditions could be strengthened by evidence that systemic inflammation [C-reactive protein ≥2 mg/L] was associated with a considerable health burden.

**Methods and results:**

A retrospective observational cohort study of 179 261 individuals in mutually exclusive cohorts (ASCVD only, CKD stages 3–4 only, ASCVD + CKD stages 3–4) and a reference cohort (no ASCVD or CKD) with ≥1 C-reactive protein measurement after diagnosis. Outcomes included all-cause mortality, healthcare resource utilization (HCRU), and direct healthcare costs. Chronic kidney disease stages 3–4, ASCVD only, and ASCVD + CKD stages 3–4 cohorts compared with the reference cohort were associated with increasing all-cause mortality (death/100 000 person-years) of 1637, 2090, 5163, and 946, respectively. HCRU events per person per year (PPPY) and mean direct costs PPPY were 20.4, 21.4, 34.2, and 8.2 and £2593, £3098, £6810, and £1144, respectively. Within cohorts, all-cause mortality increased progressively across C-reactive protein quartiles (Q) and was highest in Q4 among the ASCVD + CKD stages 3–4 cohort, with a hazard ratio (Q4 vs. Q1) of 2.75 in this cohort compared with 1.22 in the reference cohort.

**Conclusion:**

While the clinical impact of targeting inflammation is being evaluated in ongoing trials, this real-world study demonstrates that assessing inflammation in patients with ASCVD and CKD stages 3–4 can help to identify a population with a high healthcare burden.

## Graphical Abstract

**Graphical Abstract:**
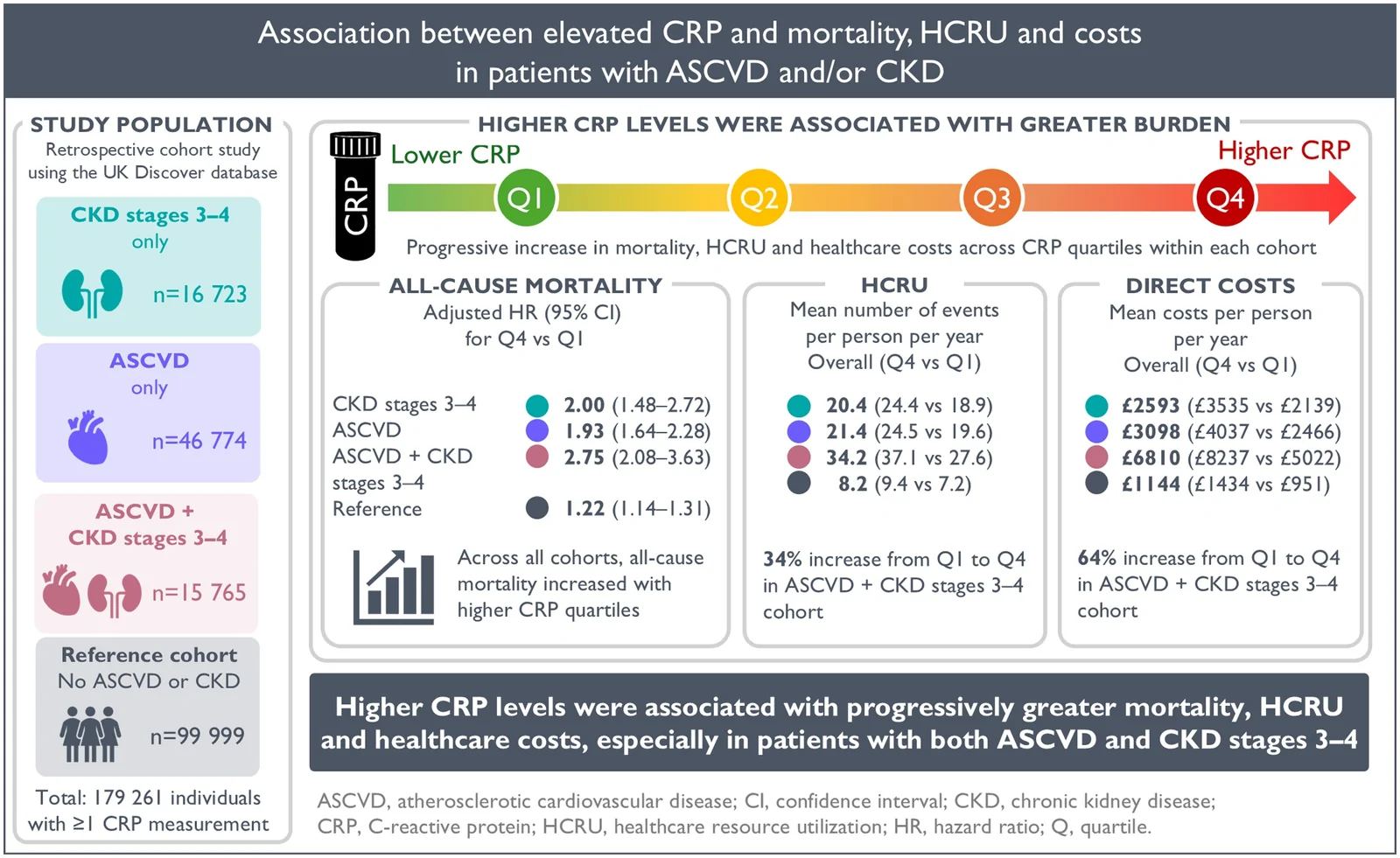

## Introduction

Cardiovascular disease (CVD) is the leading cause of death worldwide, with mortality ranging between 82.4 and 214.6 per 100 000 individuals across countries in Western Europe in 2021 and 118.1 per 100 000 individuals in the UK in 2021.^1,2^ Atherosclerotic cardiovascular disease (ASCVD) is the leading cause of CVD mortality.^3^ The interplay between genetic vulnerability and multiple risk factors over time results in the initiation and progression of atherosclerosis and its clinical manifestations such as fatal or non-fatal cardiovascular (CV) events, including myocardial infarction (MI) and ischaemic strokes.^4,5^ Chronic kidney disease (CKD) is common in people with ASCVD, with approximately one-third of people with ASCVD also having CKD.^6^ Moreover, CKD is recognized as a high-risk condition in its own right for the development of ASCVD.^7^ These two conditions also share common risk factors, such as hypertension and type 2 diabetes (T2D).^4^ Having one condition substantially increases the risk of developing the other condition,^4,8,9^ and the coexistence of both ASCVD and CKD amplifies the overall CV risk beyond the already high risk associated with each condition individually.^7,10^

Inflammation, indicated by elevated levels of C-reactive protein (≥2 mg/L), is associated with incident ASCVD and recurrent CV events among those with ASCVD,^11–13^ and is also implicated in the initiation and progression of CKD.^14,15^ The presence of inflammation, even when risk factors appear to be controlled, can be used to identify individuals who are at risk of CV events.^16–18^ Elevated high-sensitivity C-reactive protein has been associated with heart failure, peripheral artery disease, and stroke.^19–22^ Furthermore, increased C-reactive protein is associated with all-cause and CV mortality.^23–25^ Given the potential causative role of inflammation in secondary CV events, there is considerable interest in the development of therapies that target inflammation.

In addition to clinical benefits, any future approved treatments targeting inflammation and CV events could potentially bring wider benefits to healthcare systems in terms of reducing healthcare resource utilization (HCRU) and direct healthcare costs. Recent studies from a Danish national registry and US veteran populations have demonstrated that elevated C-reactive protein (≥ 2 mg/L) is associated with increased mortality, CV events, HCRU, and healthcare costs among individuals with both ASCVD and CKD.^26–28^ However, less is known about how inflammatory burden relates to outcomes across mutually exclusive ASCVD and CKD populations and how healthcare burden varies across graded levels of C-reactive protein.

In this analysis we sought to assess the possible impact of elevated C-reactive protein in individuals with ASCVD and/or CKD. We used UK real-world data to (i) examine the association between C-reactive protein levels and mortality and CV events in cohorts with ASCVD, CKD stages 3–4, and both conditions and (ii) evaluate how HCRU and costs vary between cohorts and by C-reactive protein levels within these cohorts.

## Methods

### Study ethics

This study was exempt from institutional review board approval because it was a retrospective analysis of secondary de-identified data not considered to be human subject research.

### Data source and study design

This was a retrospective observational cohort study using the Discover database, an electronic health record research dataset covering ∼2.8 million individuals living in North West London, UK, accounting for more than 95% of the North West London population.^29^ Pseudo-anonymized patient-level data are available for research through an application process via the North West London Information Governance Steering Group; this study was conducted under approval ID-135. In the Discover database, primary care data are available from 1 January 2004 onwards and linkage to secondary care data are available from 1 January 2015.

### Study population and definitions

Mutually exclusive clinical cohorts (CKD stages 3–4 only, ASCVD only, ASCVD + CKD stages 3–4) were defined based on medical records. Cohorts included adults (≥18 years old) with the presence or absence of prespecified medical conditions (ASCVD and/or CKD stages 3–4) who had at least one C-reactive protein measurement recorded after the first diagnosis of each condition. For individuals in the ASCVD + CKD stages 3–4 cohort, C-reactive protein measurements were recorded after both conditions had been diagnosed. Diagnosis of CKD stage 5 was excluded as this is a patient group with considerable mortality and HCRU, which could bias the results. A reference cohort was randomly sampled from those who met case definitions: ∼100 000 adults aged ≥50 years with no recorded diagnosis of ASCVD or CKD stages 3–5 during the study period. The age threshold for the reference cohort was selected to account for the expected high mean age of the other cohorts. See *Figure 1* for the study design.

**Figure 1:**
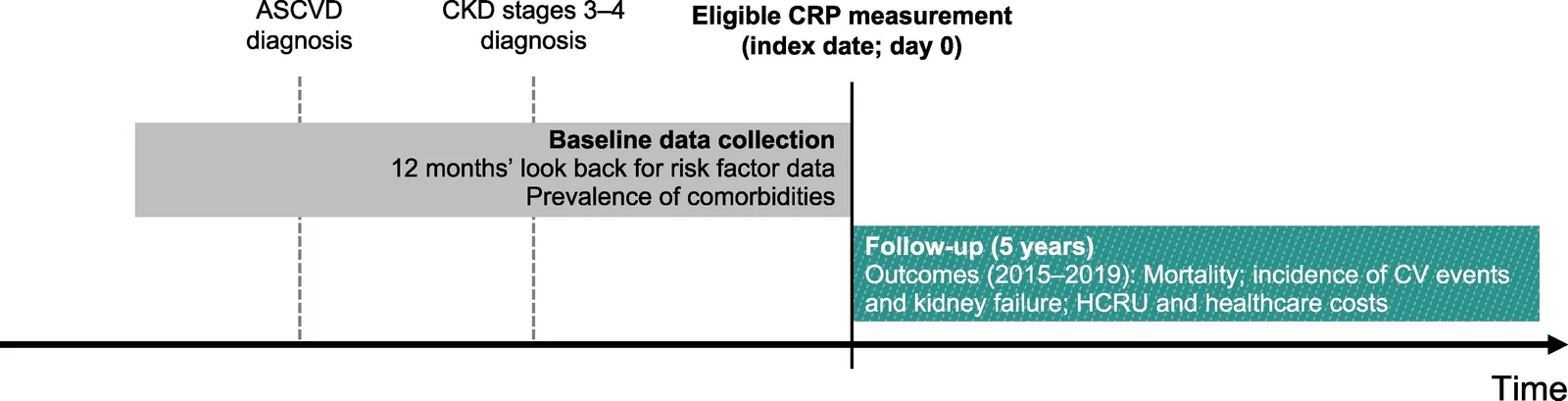
Study design. ASCVD, atherosclerotic cardiovascular disease; CKD, chronic kidney disease; CRP, C-reactive protein; CV, cardiovascular; HCRU, healthcare resource utilization.

The index date was defined as the date of the first eligible C-reactive protein measurement after all other inclusion criteria had been met. For the reference cohort, individuals with multiple C-reactive protein measurements ≤20 mg/L had only their first eligible measurement retained, with subsequent values excluded. Measurements of C-reactive protein >20 mg/L were excluded from the analyses to minimize the risk of including individuals with acute infections or autoimmune diseases. All C-reactive protein measurements were captured in primary care records, either through direct general practitioner test requests or from tests conducted in other care settings. The assays used varied, limiting the ability to standardize results.

Relevant conditions were identified using Read code information in the primary care data and International Classification of Diseases Tenth Revision (ICD-10) codes for hospital visits, outpatient appointments, and accident/emergency department attendances. The ASCVDonly cohort comprised individuals with a diagnosis of cerebrovascular disease (ICD-10 codes I63–I69), ischaemic heart disease (ICD-10 codes I20–I25), or peripheral artery disease (ICD-10 code 170), with no history of CKD stages 3–4 (ICD-10 codes N18.3 and N18.4) before cohort entry or during follow-up. The CKD stages 3–4 only cohort comprised individuals with a diagnosis of CKD stages 3–4 (ICD-10 codes N18.3 and N18.4), with no history of ASCVD before cohort entry or during follow-up. The ASCVD + CKD stages 3–4 cohort comprised individuals with documented diagnoses of both ASCVD and CKD stages 3–4 (as defined above) during the study period. The individuals who were included were followed up from the index date until death, date of record of transfer out of the region, or the end of follow-up (31 December 2019 to exclude healthcare data during the Covid-19 pandemic), whichever occurred first.

### Outcomes

Outcomes of interest were assessed between 2015 and 2019 in the predefined cohorts and also analysed by C-reactive protein quartiles (quartiles 1–4 [Q1–Q4]) within the cohorts. C-reactive protein quartiles were derived from the combined population of all four cohorts. Q1 was the lowest C-reactive protein quartile and Q4 the highest. All outcomes were age standardized using the 2013 European Standard Population.^30^

### Clinical outcomes

The clinical outcome of interest for all cohorts was all-cause mortality (defined as death recorded in primary care records—Read code or status of ‘Dead’ captured in the Whole Systems Integrated Care dataset).^31^ In addition, other outcomes were assessed in relevant cohorts: CV events (MI, coronary revascularization, stroke, and hospitalization for heart failure) were assessed in the ASCVD cohort and the ASCVD + CKD stages 3–4 cohort, and kidney failure was assessed in the CKD cohort and the ASCVD + CKD stages 3–4 cohort. Each individual was limited to one event in any given 28-day period when calculating cause-specific incidence for recurrent events (i.e. acute MI, coronary revascularization, hospitalization for acute/unstable angina, stroke, and acute heart failure). All other incident outcomes were considered non-recurrent, with individuals contributing exposure time until the first record of the event; the number of events in the study period was considered the numerator.

### Healthcare and cost-related outcomes

Annual per person per year (PPPY) HCRU events and direct costs between 2015 and 2019 were assessed. HCRU comprised emergency department visits, outpatient appointments, inpatient admissions, and primary care, while prescription counts were evaluated separately. Direct costs were defined as those associated with the aforementioned types of HCRU and prescribed medicines. The prescription cost analysis dataset in the England in 2019 was used to estimate medication costs.^32^ The mean cost across all drugs (£8.11) was assigned when actual prescription costs were missing from the dataset.^33^

### Statistical analyses

Baseline characteristics were analysed using descriptive statistics. Incidence, mortality, HCRU, and associated costs were estimated; 95% confidence intervals (CIs) were presented for mortality and HCRU. Incidence of mortality, CV events, and kidney failure were estimated by identifying the number of cause-specific events during the study period as the numerator and the sum of person-years (PYs) across each population cohort as the denominator.

Regression models were used to assess the association between C-reactive protein quartiles and outcomes in the reference, CKD stages 3–4, ASCVD, and ASCVD + CKD stages 3–4 cohorts. Cox proportional hazards regression models were utilized to evaluate the association between C-reactive protein quartiles and time to all-cause mortality and incident CV events, with the lowest C-reactive protein quartile serving as the reference quartile. Covariates included in the models were age, atrial fibrillation, cancer, chronic respiratory disease, ethnicity, heart failure, hypertension, index of multiple deprivation, MI, peripheral vascular disease, revascularization (coronary or arterial), rheumatoid diseases, sex, smoking status, stroke, and T2D. These covariates were demographic factors that are known risk factors for CV events and clinically relevant comorbidities. To identify predictors of mortality in the ASCVD + CKD stages 3–4 cohort, an additional Cox proportional hazards model was utilized to evaluate the association between covariates and all-cause mortality. All associations estimated with Cox proportional hazards models are reported as hazard ratios (HRs) and 95% CIs.

A Poisson regression model was utilized to assess the associations between C-reactive protein quartiles and HCRU events, with the lowest C-reactive protein quartile serving as the reference quartile. Covariates included the same variables as the Cox models. All associations estimated with Poisson regression models are reported as PPPY and 95% CIs. All analyses were conducted using SQL and *R* > 4.1.0.

## Results

### Study population and characteristics

In total, 179 261 individuals were included in the study. Of these, 16 723 had CKD stages 3–4 only, 46 774 had ASCVD only, 15 765 had ASCVD + CKD stages 3–4, and 99 999 were included in the reference cohort. Baseline characteristics varied across cohorts (*Table 1*). Mean age ranged from 62.1 years (standard deviation 9.9) in the reference cohort to 75.1 (9.7) years in the ASCVD + CKD stages 3–4 cohort, and the proportion of women ranged from 42.6% in the ASCVD cohort to 64.0% in the CKD stages 3–4 cohort. Mean body mass index, mean glycated haemoglobin (HbA_1c_), and mean systolic and diastolic blood pressure were similar but median lipid levels varied across all cohorts (*Table 1*). Median C-reactive protein levels were highest in the ASCVD + CKD stages 3–4 cohort (4.0 mg/L [interquartile range: 2.0–7.0]) and lowest in the reference cohort (2.6 mg/L [1.1–5.0]); intermediate values were observed in the ASCVD cohort (3.0 mg/L [1.5–5.7]) and the CKD stages 3–4 cohort (3.8 mg/L [1.9–6.1]). C-reactive protein quartiles were derived from the combined cohorts and defined as Q1: C-reactive protein 0.0–<1.4 mg/L, Q2: C-reactive protein 1.4–<3.0 mg/L, Q3: C-reactive protein 3.0–<5.0 mg/L, and Q4: C-reactive protein 5.0–20.0 mg/L. The ASCVD + CKD stages 3–4 cohort had the highest prevalence of individuals with C-reactive protein ≥2 mg/L (73.1%), followed by the CKD stages 3–4 cohort (71.3%) (*Table 1*). Comorbidities such as atrial fibrillation, hyperlipidaemia, hypertension, and T2D, were most prevalent in the ASCVD + CKD stages 3–4 cohort and least prevalent in the reference cohort (*Table 1*). Baseline characteristics per quartile are reported in Supplementary data online, *Table S1*.

**Table 1:** Baseline characteristics

Characteristic	Cohort			
	Reference (*n* = 99 999)	Individuals with CKD stages 3–4 (*n* = 16 723)	Individuals with ASCVD (*n* = 46 774)	Individuals with ASCVD + CKD stages 3–4 (*n* = 15 765)
Age, years, mean (SD)	62.1 (9.9)	68.5 (13.0)	62.2 (13.0)	75.1 (9.7)
Sex, *n* (%)				
Men	36 647 (36.6)	6020 (36.0)	26 840 (57.4)	7980 (50.6)
Women	63 351 (63.4)	10 703 (64.0)	19 934 (42.6)	7785 (49.4)
BMI, kg/m^2^, mean (SD)^a^	27.7 (5.8)	28.1 (6.0)	27.7 (5.7)	27.6 (5.6)
Smoking status—smoker, *n* (%)^b^	9109 (9.1)	1122 (6.7)	5775 (12.3)	1006 (6.4)
Ethnicity, *n* (%)^c^				
Asian	27 015 (27.0)	3676 (22.0)	13 916 (29.8)	4065 (25.8)
Black	9264 (9.3)	1924 (11.5)	3058 (6.5)	1202 (7.6)
Mixed	2297 (2.3)	376 (2.2)	840 (1.8)	293 (1.9)
Other	7876 (7.9)	809 (4.8)	2865 (6.1)	685 (4.3)
White	52 817 (52.8)	9558 (57.2)	25 292 (54.1)	9162 (58.1)
Laboratory measurements				
C-reactive protein ≥2 mg/L, *n* (%)	59 176 (67.6)	7765 (71.3)	21 114 (68.1)	6230 (73.1)
C-reactive protein, mg/L, median (IQR)	2.6 (1.1–5.0)	3.8 (1.9–6.1)	3.0 (1.5–5.7)	4.0 (2.0–7.0)
HDL-C, mmol/L, median (IQR)^d^	1.4 (1.2–1.7)	1.4 (1.1–1.7)	1.3 (1.0–1.5)	1.2 (1.0–1.5)
LDL-C, mmol/L, median (IQR)^e^	3.0 (2.4–3.7)	2.6 (2.0–3.3)	2.2 (1.7–2.9)	2.1 (1.6–2.6)
Triglycerides, mg/dL, median (IQR)^f^	106.3 (78.8–149.7)	112.5 (83.3–155.9)	107.2 (79.7–150.6)	114.3 (82.4–157.7)
HbA_1c_, %, mean (SD)^g^	6.2 (1.1)	6.5 (1.3)	6.6 (1.4)	6.8 (1.4)
Systolic blood pressure, mmHg, mean (SD)^h^	131.9 (15.9)	133.8 (16.4)	131.9 (16.5)	133.0 (18.1)
Diastolic blood pressure, mmHg, mean (SD)^i^	78.3 (9.6)	75.7 (10.0)	75.5 (10.0)	72.3 (10.7)
Comorbidities (age standardized), %				
Asthma	13.6	15.9	16.8	16.2
Atrial fibrillation	3.5	3.7	8.4	13.3
Cancer	4.7	3.8	4.2	4.5
Complex PCI	0.0	0.0	0.9	1.0
Coronary or arterial revascularization	0.0	0.0	21.8	30.8
CABG	0.0	0.0	7.2	8.7
CABG and PCI	0.0	0.0	19.6	27.1
Depression	6.6	6.8	8.0	6.9
Gout	4.2	9.7	5.1	17.1
Hyperlipidaemia	19.7	20.2	24.7	36.1
Hypertension	38.5	58.0	50.2	79.5
Ischaemic heart disease	0.0	0.0	59.7	68.5
MI	0.0	0.0	23.6	26.9
Peripheral artery disease	0.0	0.0	11.8	12.9
PCI	0.0	0.0	14.4	21.0
Simple PCI	0.0	0.0	14.0	20.3
T2D	13.8	18.2	23.1	37.5
Valvular heart disease	1.8	1.8	5.4	6.7

^a^BMI missingness: reference, 49.6%; CKD stages 3–4, 40.9%; ASCVD, 39.8%; ASCVD + CKD stages 3–4, 35.8%.

^b^Smoking status—smoker missingness: reference, 46.9%; CKD stages 3–4, 38.5%; ASCVD, 32.5%; ASCVD + CKD stages 3–4, 33.8%.

^c^Ethnicity missingness: reference, 0.7%; CKD stages 3–4, 2.3%; ASCVD, 1.7%; ASCVD + CKD stages 3–4, 2.3%.

^d^HDL-C missingness: reference, 21.8%; CKD stages 3–4, 21.1%; ASCVD, 15.3%; ASCVD + CKD stages 3–4, 16.4%.

^e^LDL-C missingness: reference, 29.3%; CKD stages 3–4, 30.5%; ASCVD, 23.9%; ASCVD + CKD stages 3–4, 26.8%.

^f^Triglycerides missingness: reference, 28.0%; CKD stages 3–4, 29.3%; ASCVD, 22.2%; ASCVD + CKD stages 3–4, 24.9%.

^g^HbA_1c_ missingness: reference, 49.5%; CKD stages 3–4, 43.7%; ASCVD, 40.6%; ASCVD + CKD stages 3–4, 35.4%.

^h^Systolic blood pressure missingness: reference, 22.3%; CKD stages 3–4, 6.4%; ASCVD, 6.4%; ASCVD + CKD stages 3–4, 3.2%.

^i^Diastolic blood pressure missingness: reference, 22.3%; CKD stages 3–4, 6.4%; ASCVD, 6.4%; ASCVD + CKD stages 3–4, 3.3%.

ASCVD, atherosclerotic cardiovascular disease; BMI, body mass index; CABG, coronary artery bypass grafting; CKD, chronic kidney disease; HbA_1c_, glycated haemoglobin; HDL-C, high-density lipoprotein cholesterol; IQR, interquartile range; LDL-C, low-density lipoprotein cholesterol; MI, myocardial infarction; PCI, percutaneous coronary intervention; SD, standard deviation; T2D, type 2 diabetes.

### Mortality

All-cause mortality per 100 000 PYs was 946 in the reference cohort, 1637 in the CKD stages 3–4 cohort, 2090 in the ASCVD cohort, and 5163 in the ASCVD + CKD stages 3–4 cohort. Across all cohorts, all-cause mortality increased progressively with higher C-reactive protein quartiles and C-reactive protein values (*Figure 2A*). The adjusted HR (95% CI) for Q4 vs. Q1 was 1.22 (1.14–1.31) in the reference cohort, 2.00 (1.48–2.72) in the CKD stages 3–4 cohort, 1.93 (1.64–2.28) in the ASCVD cohort, and 2.75 (2.08–3.63) for ASCVD + CKD stages 3–4 cohort (*Figure 2A*). HRs for all-cause mortality for C-reactive protein as a continuous variable (log-transformed) were 1.54 (1.40–1.70) for the ASCVD + CKD stages 3–4 cohort, 1.30 (1.16–1.45) for the CKD stages 3–4 cohort, 1.26 (1.19–1.34) for the ASCVD cohort, and 1.01 (0.99–1.04) for the reference cohort (*Figure 2B*).

**Figure 2:**
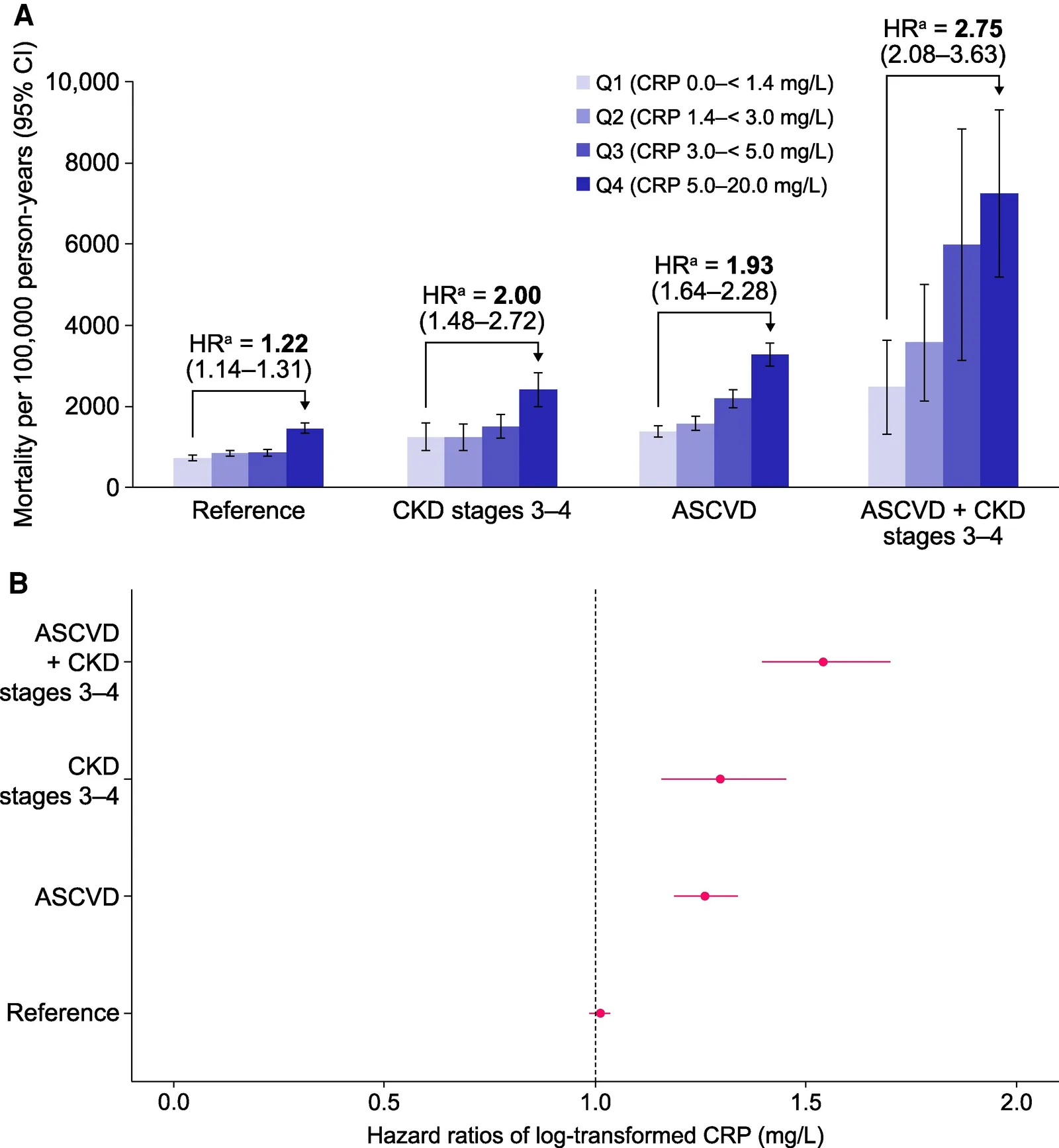
All-cause mortality assessed by C-reactive protein quartiles (*A*); all-cause mortality—HR for all cohorts (C-reactive protein combined) (*B*). ^a^HR (95% CI) for Q4 vs. Q1. HRs were adjusted for age, atrial fibrillation, cancer, chronic respiratory disease, ethnicity, heart failure, hypertension, index of multiple deprivation, MI, peripheral vascular disease, revascularization (coronary or arterial), rheumatoid diseases, sex, smoking status—smoker, stroke, and T2D. ASCVD, atherosclerotic cardiovascular disease; CI, confidence interval; CKD, chronic kidney disease; CRP, C-reactive protein; HR, hazard ratio; MI, myocardial infarction; Q, quartile; T2D, type 2 diabetes.

### Predictors of all-cause mortality in the ASCVD + CKD stages 3–4 cohort

The top predictors of all-cause mortality in the ASCVD + CKD stages 3–4 cohort were higher C-reactive protein levels, cancer, liver disease, and heart failure (*Figure 3*). The corresponding HRs (95% CI) were 2.72 (2.06–3.58) for C-reactive protein Q4 vs. Q1, 1.99 (1.61–2.47) for cancer, 1.84 (1.07–3.17) for liver disease, and 1.69 (1.40–2.04) for heart failure (*P* < 0.05).

**Figure 3:**
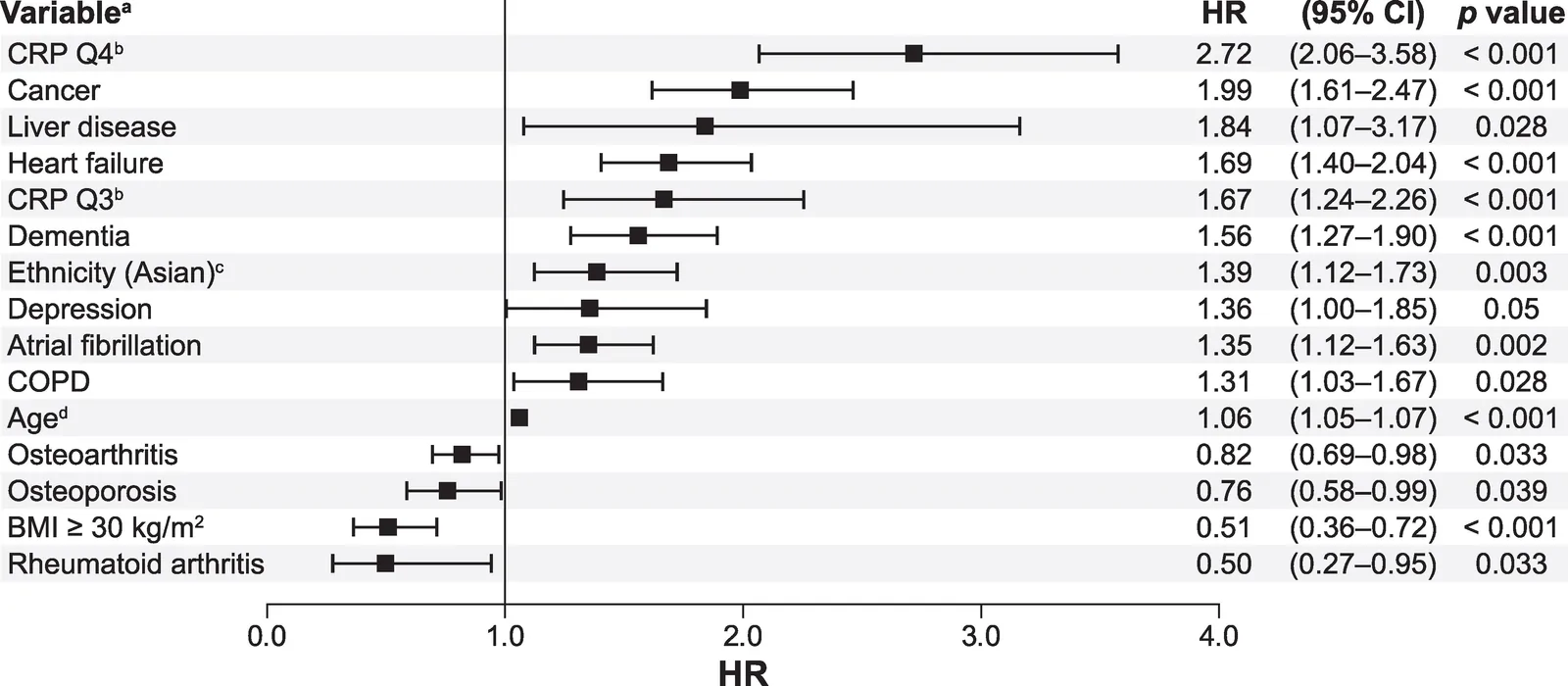
Association between covariates and all-cause mortality in the ASCVD + CKD stages 3–4 cohort, assessed by Cox proportional hazards model. ^a^For comorbidities, the reference is the absence of each comorbidity. ^b^Reference is Q1. ^c^Reference is White ethnicity. ^d^Per 10 year increase. HRs were adjusted for selected demographics, risk factor measurements and comorbidities. ASCVD, atherosclerotic cardiovascular disease; BMI, body mass index; CI, confidence interval; CKD, chronic kidney disease; COPD, chronic obstructive pulmonary disease; CRP, C-reactive protein; HR, hazard ratio; Q, quartile.

### Cardiovascular events and kidney failure

Incidence of CV events in the ASCVD and ASCVD + CKD stages 3–4 cohorts and the incidence of kidney failure in the CKD stages 3–4 and ASCVD + CKD stages 3–4 cohorts are reported in Supplementary data online, *Figure S1* and Supplementary data online, *Figure S2*, respectively. The incidence of coronary revascularization was higher than that of other CV events in the ASCVD cohort, and the incidence of acute heart failure was higher than that for other CV events in the ASCVD + CKD stages 3–4 cohort. The incidence of kidney failure was higher in the ASCVD + CKD stages 3–4 cohort than in the CKD stages 3–4 cohort. Incidence of CV events and kidney failure assessed by Creactive protein quartiles are reported in Supplementary data online, *Figure S1* and Supplementary data online, *Figure S2*, respectively. The incidence of each CV event was highest in C-reactive protein Q4 in the ASCVD cohort, except for coronary revascularization. In the ASCVD + CKD stages 3–4 cohort, the incidence of MI and coronary revascularization were highest in Creactive protein Q4. The incidence of kidney failure was highest in the ASCVD + CKD stages 3–4 Q4 cohort.

### Healthcare resource utilization

The mean number of HCRU events PPPY was lowest in the reference cohort (8.2), higher in the CKD stages 3–4 (20.4) and ASCVD (21.4) cohorts, and highest in the ASCVD + CKD stages 3–4 cohort (34.2) (*Figure 4*). Primary care events were the main driver of HCRU events PPPY across all cohorts (*Figure 4*). In general, HCRU increased progressively across C-reactive protein quartiles within each cohort (*Figure 4*). From C-reactive protein Q1 to Q4, total PPPY HCRU events increased by 25% in the ASCVD cohort, 29% in the CKD stages 3–4 cohort, 30% in the reference cohort, and 34% in the ASCVD + CKD stages 3–4 cohort. The incidence of HCRU events in the lowest C-reactive protein quartile of the ASCVD + CKD stages 3–4 cohort exceeded that observed in the highest Creactive protein quartile of all other cohorts (*Figure 4*). Chronic disease (e.g. cancer, chronic obstructive pulmonary disease, heart failure, or liver disease) was a major driver of HCRU events in the ASCVD + CKD stages 3–4 cohort (see Supplementary data online, *Figure S3*).

**Figure 4:**
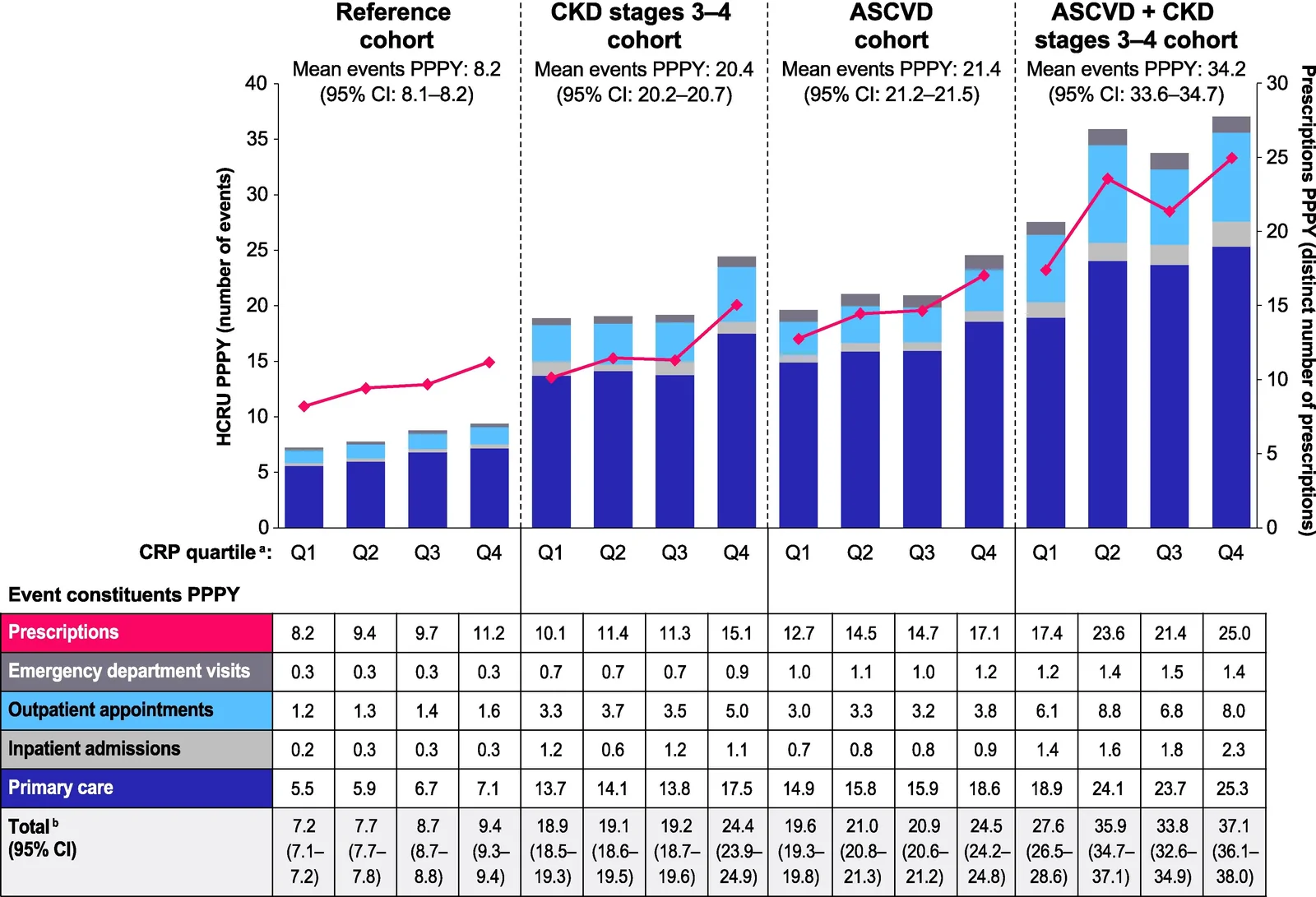
Mean PPPY HCRU and mean distinct prescriptions by C-reactive protein quartile and across cohorts. ^a^For all cohorts, CRP quartile ranges were 0.0–1.4 mg/L for Q1; >1.4–3.0 mg/L for Q2; >3.0–5.0 mg/L for Q3; and >5.0–20.0 mg/L for Q4. ^b^Total events may differ from the sum of the constituents due to rounding. Prescriptions were not included in the total events. ASCVD, atherosclerotic cardiovascular disease; CI, confidence interval; CKD, chronic kidney disease; HCRU, healthcare resource utilization; PPPY, per person per year; Q, quartile.

### Costs

Mean PPPY direct healthcare costs were highest in the ASCVD + CKD stages 3–4 cohort (£6810) and lowest in the reference cohort (£1144) (*Figure 5*). Inpatient admissions were the main driver of direct costs in all clinical cohorts, whereas prescription costs were the largest contributor in the reference cohort (see Supplementary data online, *Table S2****)***. In general, costs increased progressively across C-reactive protein quartiles within each cohort (*Figure 5* and Supplementary data online, *Figure S4*). Total PPPY costs from C-reactive protein Q1 to Q4 increased by 51% in the reference cohort; 65% in the CKD stages 3–4 cohort; 64% in the ASCVD cohort; and 64% in the ASCVD + CKD stages 3–4 cohort. Costs from C-reactive protein Q1 of the ASCVD + CKD stages 3–4 cohort exceeded those observed in C-reactive protein Q4 of all other cohorts.

**Figure 5:**
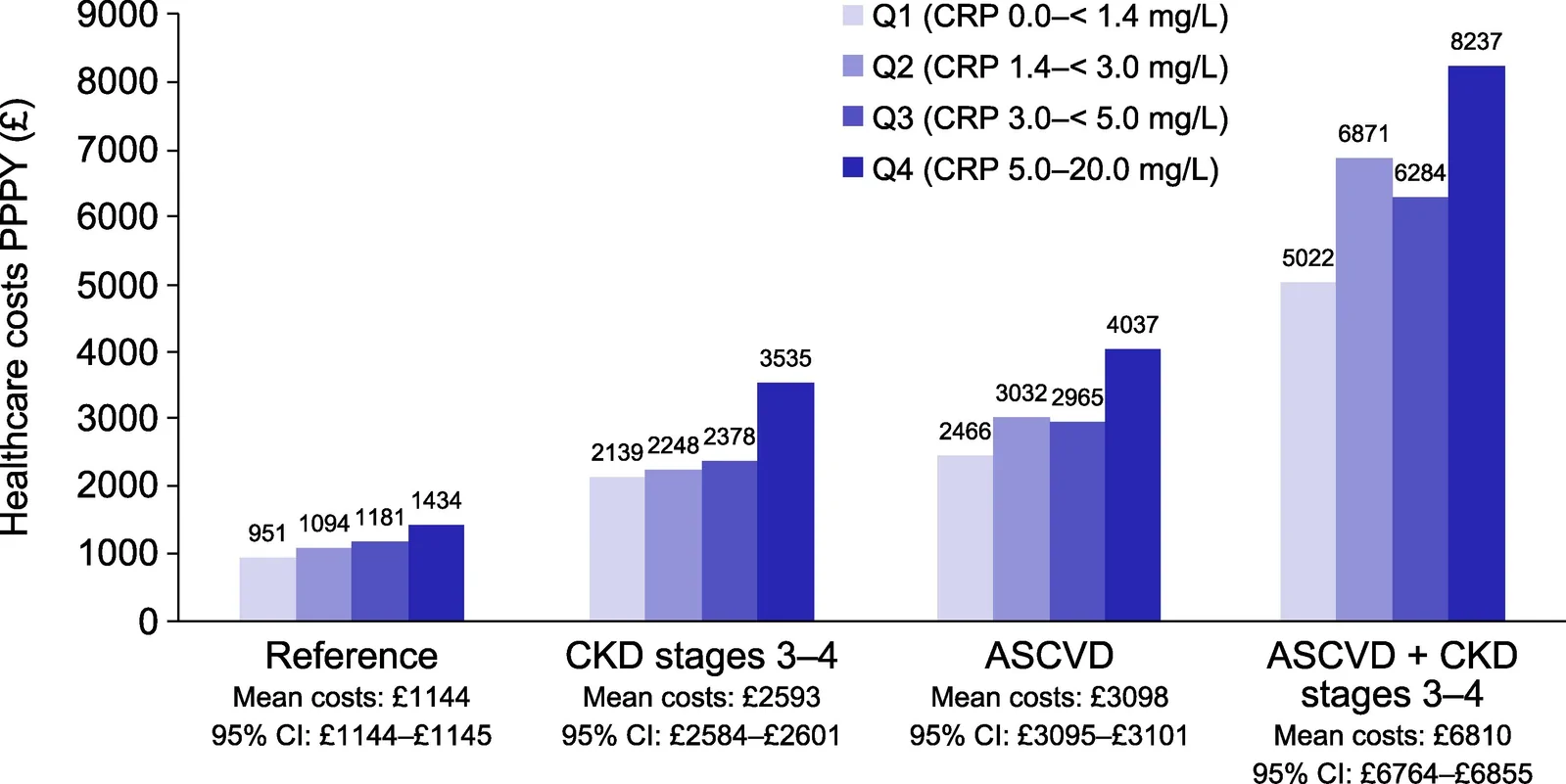
Mean PPPY healthcare costs byC-reactive protein quartile and across cohorts. ASCVD, atherosclerotic cardiovascular disease; CI, confidence interval; CKD, chronic kidney disease; CRP, C-reactive protein; PPPY, per person per year; Q, quartile.

## Discussion

In this large real-world study of individuals with ASCVD, CKD stages 3–4, or both conditions, we observed a consistent and graded association between higher C-reactive protein levels and higher risks of all-cause mortality, CV events, kidney failure, HCRU, and direct healthcare costs. Our findings are consistent with previous evidence linking higher C-reactive protein levels with adverse clinical and economic outcomes in patients with ASCVD and CKD.^26–28^ By applying a common analytical framework across mutually exclusive ASCVD only, CKD only, and ASCVD + CKD cohorts, we extend this evidence by describing how the association between C-reactive protein and outcomes varies across these populations. The ASCVD + CKD stages 3–4 group experienced the highest absolute risks and the steepest gradient across C-reactive protein quartiles, highlighting a population with a considerable unmet clinical need. Individuals with the highest C-reactive protein levels had the highest risk of all-cause mortality, most noticeable in those with both ASCVD and CKD stages 3–4 compared with either condition alone. Furthermore, HCRU and costs were generally highest in individuals with the highest C-reactive protein levels, particularly in those with ASCVD and CKD stages 3–4.

The robust association between C-reactive protein and mortality across all cohorts aligns with prior epidemiological and secondary prevention studies, including the Women’s Health Study^34^ and other analyses linking high-sensitivity C-reactive protein to incident CV events and mortality.^16,23^ Our study provides further insights by demonstrating that elevated C-reactive protein identifies individuals with ASCVD and CKD who experience not only higher clinical risk but also higher HCRU and costs. Notably, in the cohort with ASCVD + CKD stages 3–4, HCRU and costs in C-reactive protein Q1 exceeded those in Q4 of all other cohorts, suggesting an interaction between inflammation and multimorbidity.

Our findings support observations from contemporary secondary prevention datasets showing that inflammatory risk remains prognostically important despite widespread statin use.^18,19^ Even in the absence of traditional risk factors, higher levels of high-sensitivity C-reactive protein can be associated with an increased risk of coronary heart disease.^17^

Our results suggest that elevated C-reactive protein identifies subgroups with a particularly high clinical and economic burden. Although C-reactive protein is not currently established as a routine risk-stratification tool in patients with ASCVD in clinical practice, there is a shift towards high-sensitivity C-reactive protein screening to identify individuals with an unmet need for primary prevention (high-sensitivity C-reactive protein >3 mg/L) who are at increased inflammatory risk, as recommended in a scientific statement by the American College of Cardiology (ACC).^35^ Among individuals who have experienced a CV event and require secondary prevention, and who are receiving statins, the ACC recommends considering a higher statin dose adjustment if high-sensitivity C-reactive protein levels remain >2 mg/L, irrespective of low-density lipoprotein cholesterol. Similarly, the European Society of Cardiology acknowledges high-sensitivity C-reactive protein >2 mg/L as a risk modifier that may warrant reclassifying the risk category of a primary prevention individual to a higher category.^36^ That said, in Europe, high-sensitivity C-reactive protein is not routinely measured to guide risk stratification. Recommending universal screening for high-sensitivity C-reactive protein would be an expensive undertaking. As conditions such as obesity, T2D, and hypertension are independently associated with elevated hsCRP levels and increased CV risk, C-reactive protein levels provide risk prediction utility and may be feasible to use in health resource-limited settings to identify who should be prioritized for C-reactive protein testing.^37,38^ Across all covariates, Q4 C-reactive protein was the strongest predictor of mortality. Our findings suggest that elevated C-reactive protein levels, particularly in individuals with ASCVD and CKD, can help to identify high-risk individuals with a high healthcare cost burden, beyond clinical characteristics, thus highlighting an unmet need. Novel therapies that target inflammation are currently being tested in individuals with ASCVD.^39^ Moreover, the recent ACC Scientific Statement on Inflammation and Cardiovascular Disease identified targeted anti-inflammatory interventions as a strategic priority in ASCVD care.^35^

The inflammatory cascade includes the NLRP3-inflammasome IL-1β/IL-18-IL-6-C-reactive protein pathway. While targeting IL-1β has shown CV benefits in a population with prior MI, the recent press release from the ZEUS trial reported that IL-6 inhibition in patients with ASCVD and CKD did not reduce CV events.^13,40^ The IL-6 biological pathway is being investigated in other populations, with different therapies targeting IL-6 and the NLRP3 inflammasome.^39,41,42^ Colchicine is an anti-inflammatory agent included in secondary prevention guidelines and is currently the only approved therapy for this use.^35^

The strengths of our study include the use of the Discover database, which captures more than 95% of the population of North West London and links primary and secondary care records for a large and ethnically diverse population. Women accounted for 49.4% of the ASCVD + CKD stages 3–4 cohort, and 39.6% of this cohort were of non-White ethnicity. This is relevant because comparable real-world analyses of inflammation in this setting have been conducted in predominantly male or predominantly White populations.^26,43^ The open cohort study design, which allowed individuals to enter the population at any point during the study period, resulted in a large study population and reduced the risk of selection bias. The inclusion of a reference cohort without ASCVD or CKD allowed for an internal comparison. The prevalence of C-reactive protein ≥2 mg/L was similar across cohorts, at 67.6% in the reference cohort and 73.1% in the ASCVD + CKD stages 3–4 cohort. Nevertheless, log-transformed C-reactive protein was not associated with all-cause mortality in the reference cohort, but the association was strong in the cohort with both conditions. Confounding by the clinical indication for C-reactive protein testing would be expected to apply equally across all four cohorts and therefore appears unlikely to account for this contrast, although it cannot be excluded. Finally, the incorporation of various types of healthcare costs regardless of underlying causes permitted a holistic estimation of the impact of C-reactive protein levels in populations stratified by ASCVD and/or CKD or without either conditions.

The limitations of our study must also be acknowledged. Some individuals had a single C-reactive protein measurement, which may have led to misclassification of their risk stratification due to variability in C-reactive protein levels. However, there is evidence to indicate that high-sensitivity C-reactive protein levels exhibit greater variability at values >20 mg/L compared with lower values.^44^ The following individuals were excluded from our analysis, which may have led to selection bias: individuals without a CRP measurement or individuals without C-reactive protein measurements after their defining diagnoses, individuals in the ASCVD only cohort with a diagnosis code for CKD stages 3–4 during the follow-up, and individuals in the CKD stages 3–4 only cohort with a diagnosis code of ASCVD during the follow-up. Although C-reactive protein values >20 mg/L were excluded, some measurements may have been obtained near acute CV events, infections, or other inflammatory conditions. Furthermore, inflammatory diseases were not systematically excluded using diagnostic codes, raising the possibility of residual confounding from chronic inflammatory disorders. Additionally, the study may have been subject to survivorship bias as the analysis only included individuals who were followed up until death or moved out of the region. The design of the cohorts, where diagnosis codes for ASCVD and/or CKD during follow-up were used to determine cohort eligibility, may have resulted in selection bias in the ASCVD only and CKD only cohorts due to conditioning on future events. Individuals with recorded C-reactive protein measurements may differ systematically from those without C-reactive protein testing, as C-reactive protein testing is likely to occur for a specific reason in routine clinical practice. Consequently, the study population may represent individuals with greater healthcare engagement or higher burden of diseases. Additionally, there was no direct information on the assays used; therefore, the lower limit of detection might have influenced results. There was also a high percentage of missingness for some measurements at baseline (e.g. lipids, HbA_1c_) and not all prescription costs could be mapped. Where data were missing, the mean cost for a prescription in England in 2019 (£8.11) was assigned from the prescription cost analysis dataset.

## Conclusions

In conclusion, in this UK study, individuals with the highest C-reactive protein levels had the highest risk of death, most noticeably in a cohort with both ASCVD and CKD stages 3–4. Higher HCRU and costs were observed for groups with higher C-reactive protein levels within each cohort; healthcare costs were highest for individuals with both ASCVD and CKD stages 3–4, exceeding the combined costs associated with each condition alone. Individuals with ASCVD and CKD stages 3–4 plus elevated C-reactive protein levels may represent an at-risk group with a high unmet need for the development of novel therapies specifically targeting inflammation.

## Supplementary Material

oeag177_Supplementary_Data

## Data Availability

The authors confirm that all data supporting the findings of this study are available within the article and its supplementary materials. Discover does not permit sharing or publication of individual patient data. Information on how to access Discover for research purposes can be found here: https://discover-now.co.uk/how-to-access-the-data/.
