# Supplementary material for: Association between elevated C-reactive protein and mortality, healthcare resource utilization and costs in patients with atherosclerotic cardiovascular disease and/or chronic kidney disease
Source: Eur Heart J Open. 2026 Oct 14;6(6):oeag177. doi: 10.1093/ehjopen/oeag177 (PMC13756081; doi:10.1093/ehjopen/oeag177)
Supplement: Supplementary file 1 [file oeag177_supplementary_data.pdf]

Supplementary materials

Association between elevated C-reactive protein and mortality, healthcare resource utilization and costs in patients with atherosclerotic cardiovascular disease and/or chronic kidney disease

Authors:

Kausik K. Ray[1], Fotios Barkas[1,2], Eline L. Huisman[3], Jimmi Mathisen[4], Iram Faqir Muhammad[4], Hongye Ren[4], Simon Bertram Reuter[4], Jonathan Pearson-Stuttard[5,6], and Andrew Thompson[5]

Affiliations:

[1]Department of Primary Care and Public Health, School of Public Health, Imperial College London, London, UK

[2]Department of Internal Medicine, Faculty of Medicine, School of Health Sciences, University of Ioannina, Ioannina, Greece

[3]Novo Nordisk BV, Amsterdam, Netherlands

[4]Novo Nordisk A/S, Søborg, Denmark

[5]LCP Health, Lane Clark & Peacock LLP, London, UK

[6]Department of Epidemiology and Biostatistics, School of Public Health, Imperial College London, London, UK

Corresponding author: Kausik K. Ray, Department of Primary Care and Public Health, Imperial College London, 320 Reynolds Building, Charing Cross Campus, United Kingdom.

Email: k.ray@imperial.ac.uk

Supplementary Table 1 Baseline characteristics by quartile

	Reference				CKD stages 3–4				ASCVD				ASCVD + CKD stages 3–4			
**Characteristic**	**Q1**	**Q2**	**Q3**	**Q4**	**Q1**	**Q2**	**Q3**	**Q4**	**Q1**	**Q2**	**Q3**	**Q4**	**Q1**	**Q2**	**Q3**	**Q4**
N	25 539	24 798	28 564	21 098	4042	4205	4261	4215	11 593	12 728	10 789	11 664	5459	2817	3629	3860
Age, years, mean (SD)	61.8 (9.6)	62.0 (9.7)	61.9 (9.7)	62.9 (10.5)	67.4 (13.1)	68.2 (12.7)	68.6 (12.8)	69.6 (13.3)	61.9 (12.9)	62.1 (12.9)	61.7 (12.7)	63.1 (13.3)	75.1 (9.5)	74.9 (9.9)	75.0 (9.6)	75.5 (9.8)
Sex, n (%)																
Men	9954 (39.0)	9608 (38.7)	10 145 (35.5)	6940 (32.9)	1528 (37.8)	1531 (36.4)	1446 (33.9)	1515 (35.9)	7013 (60.5)	7410 (58.2)	6112 (56.7)	6305 (54.1)	2793 (51.2)	1379 (49.0)	1826 (50.3)	1982 (51.3)
Women	15 584 (61.0)	15 190 (61.3)	18 419 (64.5)	14 158 (67.1)	2514 (62.2)	2674 (63.6)	2815 (66.1)	2700 (64.1)	4580 (39.5)	5318 (41.8)	4677 (43.3)	5359 (45.9)	2666 (48.8)	1438 (51.0)	1803 (49.7)	1878 (48.7)
BMI, kg/m², mean (SD)	25.4 (4.6)	27.1 (5.1)	28.3 (5.6)	30.1 (6.7)	26.5 (5.1)	27.7 (5.5)	28.4 (6.0)	29.6 (6.8)	26.4 (4.8)	27.6 (5.2)	27.8 (5.6)	28.8 (6.6)	26.7 (5.1)	27.9 (5.6)	28.0 (5.7)	28.5 (6.1)
Smoker, n (%)	1977 (7.7)	2124 (8.6)	2703 (9.5)	2310 (10.9)	236 (5.8)	258 (6.1)	319 (7.5)	313 (7.4)	1137 (9.8)	1424 (11.2)	1427 (13.2)	1790 (15.3)	290 (5.3)	188 (6.7)	236 (6.5)	295 (7.6)
Ethnicity, n (%)																
Asian	6483 (25.4)	6707 (27.0)	8291 (29.0)	5534 (26.2)	925 (22.9)	974 (23.2)	963 (22.6)	814 (19.3)	3622 (31.2)	4130 (32.4)	3223 (29.9)	2941 (25.2)	1588 (29.1)	718 (25.5)	917 (25.3)	842 (21.8)
Black	2293 (9.0)	2211 (8.9)	2551 (8.9)	2209 (10.5)	597 (14.8)	485 (11.5)	428 (10.0)	414 (9.8)	859 (7.4)	740 (5.8)	673 (6.2)	786 (6.7)	482 (8.8)	196 (7.0)	258 (7.1)	266 (6.9)
Mixed	606 (2.4)	597 (2.4)	618 (2.2)	476 (2.3)	114 (2.8)	94 (2.2)	78 (1.8)	90 (2.1)	248 (2.1)	219 (1.7)	176 (1.6)	197 (1.7)	113 (2.1)	41 (1.5)	70 (1.9)	69 (1.8)
Other	2297 (9.0)	2023 (8.2)	2154 (7.5)	1402 (6.6)	248 (6.1)	192 (4.6)	174 (4.1)	195 (4.6)	821 (7.1)	781 (6.1)	659 (6.1)	604 (5.2)	243 (4.5)	118 (4.2)	151 (4.2)	173 (4.5)
White	13694 (53.6)	13110 (52.9)	14728 (51.6)	11285 (53.5)	2105 (52.1)	2392 (56.9)	2495 (58.6)	2566 (60.9)	5927 (51.1)	6714 (52.7)	5822 (54.0)	6829 (58.5)	2964 (54.3)	1702 (60.4)	2100 (57.9)	2396 (62.1)
Laboratory measurements																

	Reference				CKD stages 3–4				ASCVD				ASCVD + CKD stages 3–4			
**Characteristic**	**Q1**	**Q2**	**Q3**	**Q4**	**Q1**	**Q2**	**Q3**	**Q4**	**Q1**	**Q2**	**Q3**	**Q4**	**Q1**	**Q2**	**Q3**	**Q4**
CRP, mg/L, median (IQR)	1.0 (0.7–1.0)	2.0 (2.0–2.0)	4.0 (4.0–5.0)	8.7 (6.7–12.0)	1.0 (0.7–1.0)	2.0 (2.0–2.1)	4.0 (4.0–5.0)	9.0 (7.0–13.0)	1.0 (0.6–1.0)	2.0 (2.0–2.0)	4.0 (4.0–5.0)	9.0 (7.0–13.0)	1.0 (0.7–1.0)	2.0 (2.0–2.1)	4.0 (4.0–5.0)	9.8 (7.0–14.0)
HDL-C, mmol/L, mean (SD)	1.6 (0.5)	1.5 (0.4)	1.4 (0.4)	1.4 (0.4)	1.5 (0.5)	1.4 (0.4)	1.4 (0.4)	1.3 (0.4)	1.4 (0.4)	1.3 (0.4)	1.3 (0.4)	1.3 (0.4)	1.4 (0.4)	1.3 (0.4)	1.3 (0.4)	1.2 (0.4)
LDL-C, mmol/L, mean (SD)	3.0 (0.9)	3.1 (1.0)	3.1 (0.9)	3.1 (1.0)	2.6 (1.0)	2.6 (0.9)	2.8 (1.0)	2.7 (1.0)	2.2 (0.9)	2.3 (0.9)	2.4 (0.9)	2.5 (0.9)	2.1 (0.8)	2.2 (0.9)	2.3 (0.9)	2.2 (0.9)
Triglycerides, mg/dL, mean (SD)	109.7 (65.0)	123.0 (75.1)	131.5 (77.0)	136.2 (78.4)	119.0 (66.2)	129.0 (75.0)	132.2 (72.0)	138.6 (80.1)	114.2 (74.3)	127.6 (78.4)	132.1 (81.7)	137.6 (87.9)	120.9 (65.8)	134.0 (80.5)	136.7 (80.7)	139.0 (82.3)
HbA_{1c}, %, mean (SD)	6.0 (1.0)	6.1 (1.0)	6.3 (1.2)	6.4 (1.3)	6.4 (1.2)	6.4 (1.2)	6.5 (1.4)	6.6 (1.5)	6.4 (1.2)	6.5 (1.3)	6.7 (1.4)	6.8 (1.5)	6.7 (1.3)	6.8 (1.4)	7.0 (1.5)	7.0 (1.6)
Systolic blood pressure, mmHg, mean (SD)	130.4 (15.7)	131.6 (15.8)	132.5 (15.9)	133.1 (16.1)	132.9 (16.5)	133.9 (15.8)	134.7 (16.5)	133.5 (16.9)	131.2 (15.8)	131.8 (15.8)	132.7 (16.8)	131.9 (17.4)	132.8 (17.2)	133.1 (18.3)	134.1 (18.5)	132.1 (18.8)
Diastolic blood pressure, mmHg, mean (SD)	77.6 (9.5)	78.2 (9.6)	78.7 (9.5)	78.7 (9.7)	75.2 (9.8)	75.9 (9.9)	76.1 (10.1)	75.7 (10.3)	75.1 (9.6)	75.4 (9.9)	76.0 (10.0)	75.5 (10.6)	72.0 (10.4)	72.2 (10.5)	72.9 (10.9)	72.0 (11.2)
Comorbidities, (age standardized), %																
Atrial fibrillation	2.7	3.2	3.6	4.4	2.4	4.2	3.5	4.7	6.6	8.4	7.5	11.1	11.8	12.8	16.8	16.4
T2D	13.4	13.8	13.6	15.0	17.9	18.7	17.1	19.8	21.5	22.0	23.0	26.2	35.8	46.6	34.3	48.2
Hyperlipidaemia	20.5	21.0	19.0	18.3	21.3	22.1	19.1	18.9	24.3	25.1	24.4	25.1	34.1	38.8	30.2	38.5
Hypertension	35.2	37.7	39.6	42.4	55.1	57.2	57.0	63.5	47.4	49.7	51.6	53.1	82.1	81.4	77.7	84.2

For all cohorts, CRP quartile ranges were 0–1.4 mg/L for Q1; >1.4–3.0 mg/L for Q2; >3.0–5.0 mg/L for Q3; and >5.0–20.0 mg/L for Q4.

ASCVD, atherosclerotic cardiovascular disease; BMI, body mass index; CKD, chronic kidney disease; CRP, C-reactive protein; HbA1c, glycated haemoglobin; HDL-C, high-density lipoprotein cholesterol; LDL-C, low-density lipoprotein cholesterol; Q, quartile; SD, standard deviation; T2D, type 2 diabetes.

Supplementary Table 2 Healthcare cost constituents PPPY

	Reference				CKD stages 3–4				ASCVD				ASCVD + CKD stages 3–4			
Cost constituents PPPY, £	**Q1**	**Q2**	**Q3**	**Q4**	**Q1**	**Q2**	**Q3**	**Q4**	**Q1**	**Q2**	**Q3**	**Q4**	**Q1**	**Q2**	**Q3**	**Q4**
Prescriptions	350	411	416	526	501	555	561	796	634	804	814	1179	991	1841	1748	2028
Emergency department visits	28	31	34	40	71	73	76	105	121	132	123	156	159	183	187	194
Outpatient appointments	139	150	170	189	418	484	456	634	373	414	396	469	775	1078	837	1026
Inpatient admissions	293	350	390	498	796	771	928	1544	954	1274	1224	1753	2611	3143	2906	4329
Primary care	141	151	171	182	353	364	356	456	382	409	408	480	486	625	606	660
Total[a] (95% CI)	951 (950–952)	1094 (1093–1095)	1181 (1180–1182)	1434 (1433–1436)	2139 (2113–2165)	2248 (2218–2278)	2378 (2348–2407)	3535 (3490–3580)	2466 (2456–2475)	3032 (3021–3042)	2965 (2955–2976)	4037 (4019–4056)	5022 (4824–5220)	6871 (6638–7103)	6284 (6119–6448)	8237 (8091–8383)

[a]Total costs may differ from the sum of the constituents due to rounding.

For all cohorts, CRP quartile ranges were 0–1.4 mg/L for Q1; >1.4–3.0 mg/L for Q2; >3.0–5.0 mg/L for Q3; and >5.0–20.0 mg/L for Q4.

ASCVD, atherosclerotic cardiovascular disease; CI, confidence interval; CKD, chronic kidney disease; CRP, C-reactive protein; HCRU, healthcare resource utilization; PPY, per person year; Q, quartile.

Supplementary Figure 1 Incidence of CV events in the ASCVD and ASCVD + CKD stages 3–4 cohorts (A); Incidence of CV events assessed by CRP quartiles in the ASCVD and ASCVD + CKD stages 3–4 cohorts (B)

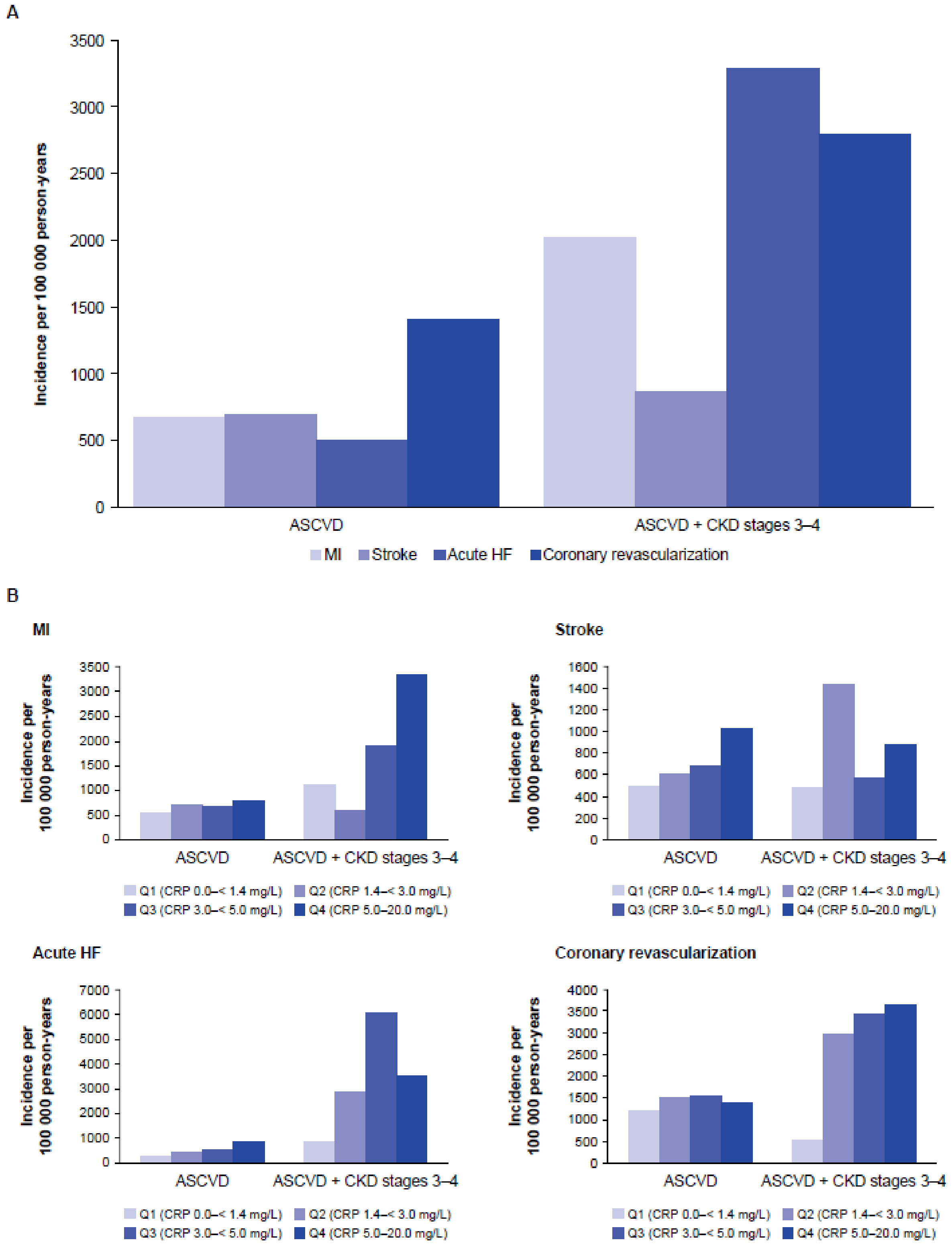

ASCVD, atherosclerotic cardiovascular disease; CKD, chronic kidney disease; CRP, C-reactive protein; CV, cardiovascular; HF, heart failure; MI, myocardial infarction; Q, quartile.

Supplementary Figure 2 Incidence of kidney failure in the CKD stages 3–4 and ASCVD + CKD stages 3–4 cohorts (A); Incidence of kidney failure assessed by CRP quartiles in the CKD stages 3–4 and ASCVD + CKD stages 3–4 cohorts

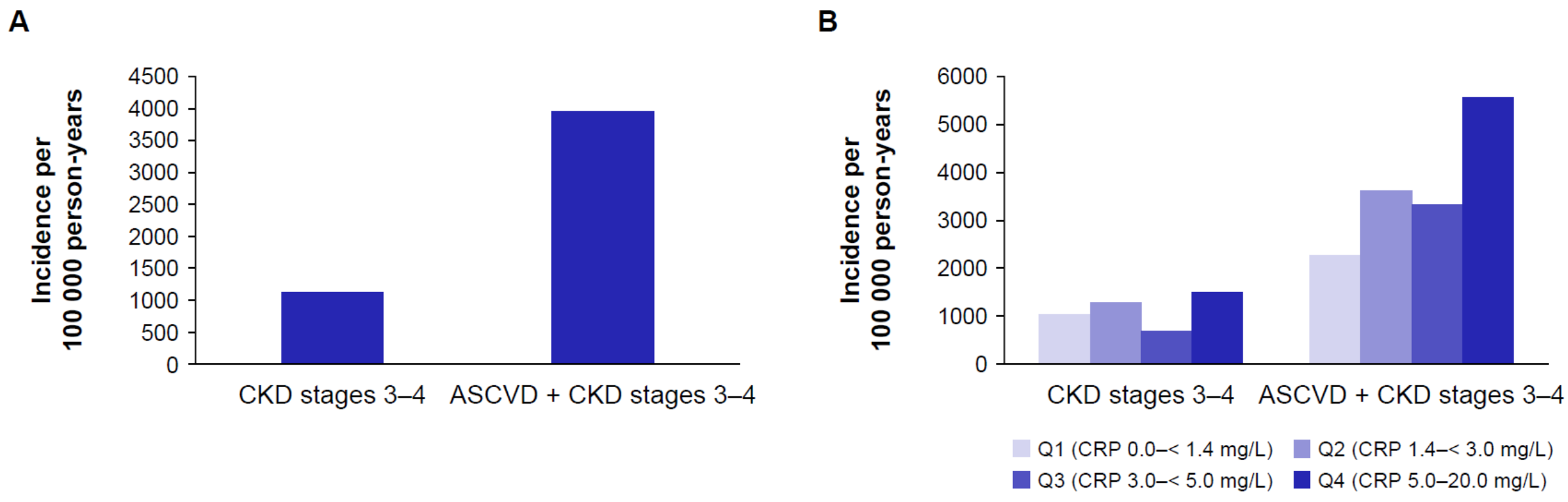

ASCVD, atherosclerotic cardiovascular disease; CKD, chronic kidney disease; CRP, C-reactive protein; Q, quartile.

Supplementary Figure 3 IRR (95% CI) of HCRU across CRP in the ASCVD + CKD stages 3–4 cohort

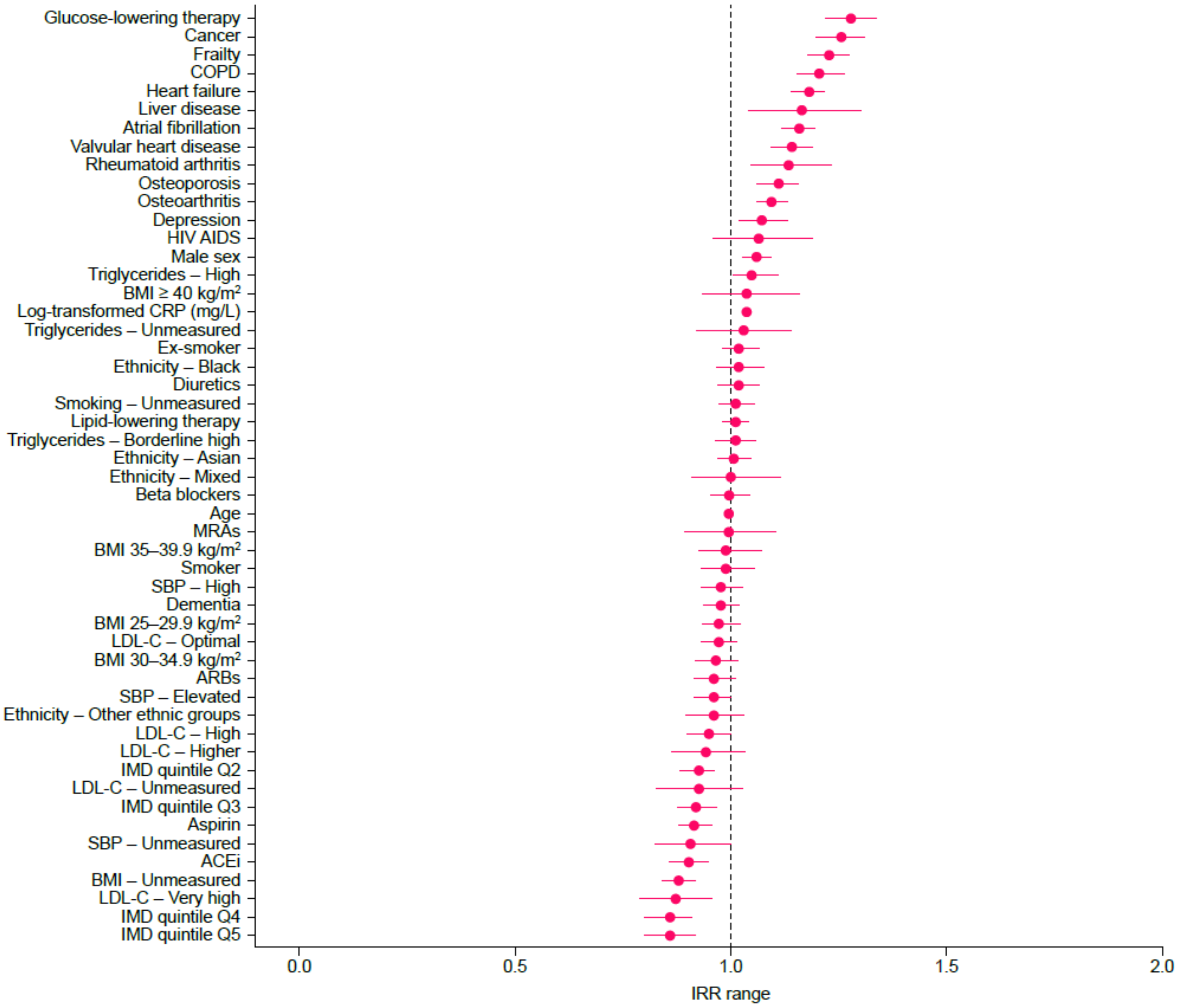

ACEi, angiotensin-converting enzyme inhibitor; AIDS, acquired immune deficiency syndrome; ARB, angiotensin receptor blocker; ASCVD, atherosclerotic cardiovascular disease; BMI, body mass index; CI, confidence interval; CKD, chronic kidney disease; COPD, chronic obstructive pulmonary disease; CRP, C-reactive protein; HCRU, healthcare resource utilization; HIV, human immunodeficiency virus; IMD, index of multiple deprivation; IRR, incidence rate ratio; LDL-C, low-density lipoprotein cholesterol; MRA, mineralocorticoid receptor antagonist; Q, quartile; SBP, systolic blood pressure.

Supplementary Figure 4 Average marginal effect for CRP

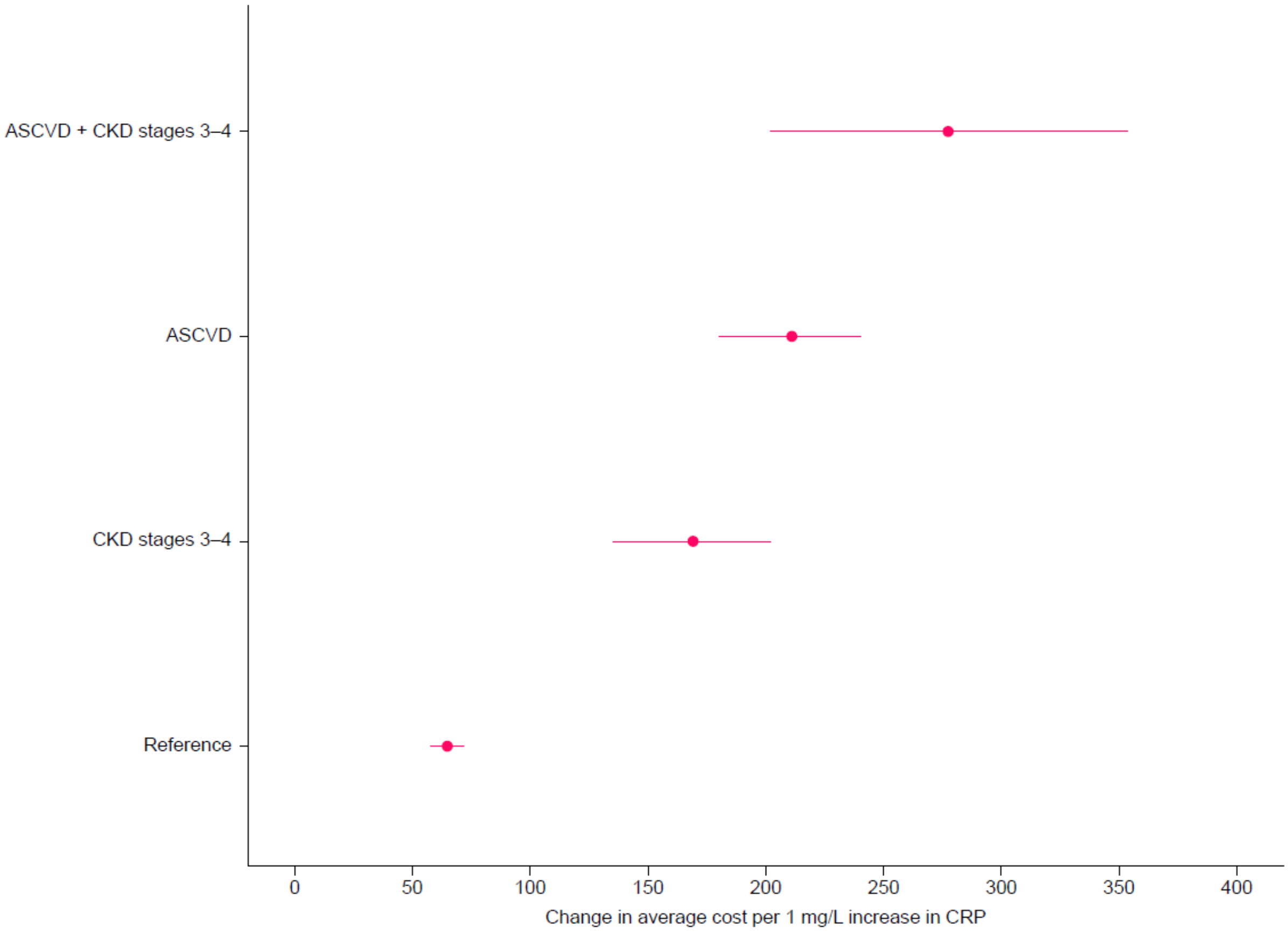

Error bars represent 95% CI.

ASCVD, atherosclerotic cardiovascular disease; CI, confidence interval; CKD, chronic kidney disease; CRP, C-reactive protein.
